# Gut microbiome and NAFLD: impact and therapeutic potential

**DOI:** 10.3389/fmicb.2024.1500453

**Published:** 2024-11-27

**Authors:** Liwei Li, Fuqing Cai, Chen Guo, Zheng Liu, Jiamin Qin, Jiean Huang

**Affiliations:** Department of Gastroenterology, The Second Affiliated Hospital of Guangxi Medical University, Nanning, China

**Keywords:** NAFLD, gut microbiome, microbial therapeutics, gut virome, gut fungi, parasites

## Abstract

Non-Alcoholic Fatty Liver Disease (NAFLD) affects approximately 32.4% of the global population and poses a significant health concern. Emerging evidence underscores the pivotal role of the gut microbiota—including bacteria, viruses, fungi, and parasites—in the development and progression of NAFLD. Dysbiosis among gut bacteria alters key biological pathways that contribute to liver fat accumulation and inflammation. The gut virome, comprising bacteriophages and eukaryotic viruses, significantly shapes microbial community dynamics and impacts host metabolism through complex interactions. Similarly, gut fungi maintain a symbiotic relationship with bacteria; the relationship between gut fungi and bacteria is crucial for overall host health, with certain fungal species such as *Candida* in NAFLD patients showing detrimental associations with metabolic markers and liver function. Additionally, the “hygiene hypothesis” suggests that reduced exposure to gut parasites may affect immune regulation and metabolic processes, potentially influencing conditions like obesity and insulin resistance. This review synthesizes current knowledge on the intricate interactions within the gut microbiota and their associations with NAFLD. We highlight the therapeutic potential of targeting these microbial communities through interventions such as probiotics, prebiotics, and fecal microbiota transplantation. Addressing the complexities of NAFLD requires comprehensive strategies that consider the multifaceted roles of gut microorganisms in disease pathology.

## Highlights

- Lytic and lysogenic bacteriophages disrupt the gut microbiota by causing bacterial lysis, which leads to the release of LPS and subsequent inflammation.
- *Candida albicans* and *Mucor ambiguus* activate NF-κB-mediated inflammation affecting insulin resistance and lipid metabolism.
- *Saccharomyces cerevisiae* and *Schizosaccharomyces pombe* improve insulin resistance by activating anti-inflammatory pathway through *β*-glucans.
- Blastocystis ST7 interacts with gut bacteria, disrupting tight junctions and increasing pro-inflammatory cytokines, thereby influencing NAFLD development.

## Introduction

Non-Alcoholic Fatty Liver Disease (NAFLD) is a common liver disorder affecting approximately 32.4% of the global population (Riazi et al., 2022). It is characterized by excessive fat accumulation in the liver, which leads to various health complications. Although the precise causes of NAFLD remain unclear, several key risk factors have been identified, including obesity, type 2 diabetes, high cholesterol, insulin resistance, and poor dietary habits (Kneeman et al., 2012; Basaranoglu et al., 2015; Mirmiran et al., 2017; Abenavoli et al., 2022). Additionally, gut microbiota dysbiosis are recognized as significant contributors to the onset and progression of NAFLD (Hrncir et al., 2021).

The human gut is a complex ecosystem that hosts a vast array of microorganisms, collectively known as the gut microbiome. This microbiome includes bacteria, viruses, fungi, and parasites, all of which coexist in a delicate equilibrium (Fay et al., 2017). Notably, the microorganisms in the gut outnumber human cells by a substantial margin, with estimates ranging between 10^13^ and 10^14^ cells—approximately 10 times more than the number of human cells (Bäckhed et al., 2005; Neish, 2009). These microorganisms also carry a genetic material that is roughly 100 times greater than the human genome. In addition to their abundance, these microorganisms produce metabolites that have significant effects on human health. The complex interactions among gut microbiota are closely linked with various metabolic disorders, and the metabolites they produce have far-reaching impacts on health (Boulangé et al., 2016; Dabke et al., 2019; Breuninger et al., 2021).

Within the gut ecosystem, the virome plays a key role. The gut virome consists of prokaryotic viruses, primarily bacteriophages, which infect bacteria, as well as eukaryotic viruses that target human gut cells, fungi, and protozoa (Cao et al., 2022). Viruses infecting prokaryotes dominate the gut virome, making up 97.8% of its composition, including bacteriophages at 97.7% and archaeal viruses at 0.1%, while eukaryotic viruses account for the remaining 2.1% (Spencer et al., 2022). The interactions between bacteriophages and bacteria regulate the gut microenvironment and have a profound influence on human health.

Gut fungi also play a crucial role in this ecosystem. They help regulate the immune system, produce metabolites that impact metabolic diseases, and influence both the composition and function of the gut microbiota (van Tilburg Bernardes et al., 2020; Pérez, 2021; Zhang F. et al., 2022). Protozoa, which can act as both beneficial commensals and potential disease-causing agents, add another layer of complexity to this finely balanced system (Chabé et al., 2017; Dubik et al., 2022). Indeed, all microorganisms in the gut—including bacteria, fungi, viruses, and protozoa—have dual roles, contributing to host health while also possessing the potential to cause disease under certain conditions.

Emerging evidence suggests that probiotics may have a beneficial effect on NAFLD by modulating the gut microbiota and reducing inflammation. Studies indicate that the administration of probiotics could help restore the balance of gut microbiota, reduce hepatic inflammation, and improve liver health by preventing gut dysbiosis (Wang L. et al., 2023). Probiotics, particularly those that target gut bacteria and improve bile acid metabolism, have been shown to alleviate hepatic steatosis and support metabolic health in patients with NAFLD (Xie and Halegoua-DeMarzio, 2019; Mims et al., 2021). The modulation of the gut-liver axis through probiotics offers a promising approach for the prevention and treatment of NAFLD (Abenavoli et al., 2022).

Additionally, noninvasive biomarkers and surrogate scores are becoming increasingly important for diagnosing and assessing the severity of NAFLD, particularly in the context of liver fibrosis. Tools such as the NAFLD Fibrosis Score (NFS), Fibrosis-4 (FIB-4), and NAFLD Fibrosis Protein Panel (NFPP) provide valuable information regarding the extent of liver damage and help guide clinical decisions (Elsayed et al., 2022). Identifying patients at risk for developing severe liver complications through these noninvasive measures could allow for early interventions that target inflammation and fibrosis, ultimately preventing the progression of NAFLD.

This review aims to thoroughly explore the multifaceted roles of the gut microbiota in relation to NAFLD. It focuses on understanding how the gut environment influences the development and progression of the disease. Maintaining a stable gut microenvironment is critical to understanding the pathogenesis of NAFLD. A detailed examination of the relationship between the gut environment and NAFLD reveals the interconnectedness of various factors contributing to the disease. By understanding the key roles played by gut microbiota—including bacteria, viruses, fungi, and parasites—researchers and healthcare professionals can gain valuable insights into the complex mechanisms driving the onset and progression of NAFLD (Mims et al., 2021; Wang L. et al., 2023). Furthermore, the identification and use of noninvasive biomarkers can enhance the early detection of disease and provide better strategies for managing NAFLD (Elsayed et al., 2022). The review emphasizes the need for further research into these intricate relationships in order to develop more effective strategies for both the prevention and treatment of NAFLD.

### Unraveling the role of gut bacteria in NAFLD

In patients with NAFLD, compelling evidence suggests significant dysbiosis in the gut bacteria, accompanied by a marked reduction in alpha diversity (Khan et al., 2021; Fang et al., 2022). NAFLD patients frequently exhibit a gut microbiota predominantly dominated by *Firmicutes*, resulting in an elevated *Firmicutes*-to-*Bacteroidetes* ratio compared to healthy controls (Hrncir et al., 2021; Aron-Wisnewsky et al., 2020; Tokuhara, 2021; Jasirwan et al., 2021). The increased abundance of *Bacteroides vulgatus*, *Escherichia coli*, and *Klebsiella pneumoniae* in NAFLD is associated with metabolic alterations, particularly in obesity and insulin resistance. *B. vulgatus* and *E. coli* promote hepatic fat accumulation and exacerbate insulin resistance by producing lipopolysaccharides (LPS), which activates the Toll-like receptor 4 (TLR4) pathway in the liver and nuclear factor kappa B (NF-κB) signaling pathways (Aron-Wisnewsky et al., 2020; Shi et al., 2006; Cai et al., 2005). Under inflammatory conditions, there is a decrease in hepatic lipase mRNA expression, along with an upregulation of the expression and activity of HMG-CoA reductase, the rate-limiting enzyme in cholesterol synthesis. As a result, lipid hydrolysis in the liver is reduced, whereas cholesterol production is increased (Feingold et al., 1993, 1999). *K. pneumoniae* increases oxidative stress and pro-inflammatory cytokine release, directly damaging hepatocytes (Aron-Wisnewsky et al., 2020). Ethanol also activates the NF-κB and apoptosis-related pathways, accelerating the progression of NAFLD to non-alcoholic steatohepatitis (NASH) (Mandrekar and Szabo, 2009). Conversely, beneficial bacteria such as *Akkermansia muciniphila*, *Faecalibacterium prausnitzii*, and *Bifidobacterium* are depleted in NAFLD patients (Aron-Wisnewsky et al., 2020). *A. muciniphila* improves intestinal barrier function by downregulating the NF-κB pathway, regulating the mucus layer, and modulating tight junction proteins, leading to reduced LPS absorption (Everard et al., 2013). Furthermore, Amuc_1100, a specific protein isolated from the outer membrane of *A. muciniphila*, can interact with Toll-like receptor 2 (TLR2), regulate various tight-junction proteins including occludin and claudin 3, contributing to the improvement of the intestinal barrier (Plovier et al., 2017). In addition, *A. muciniphila* produces short-chain fatty acids (SCFAs), which activate G protein-coupled receptors (GRPs), thereby modulating energy metabolism and providing protection against NAFLD (Kimura et al., 2014). *F. prausnitzii* produces anti-inflammatory SCFAs, particularly butyrate, which inhibits the NF-κB signaling pathway and reduces the release of pro-inflammatory cytokines (Miquel et al., 2013). Through SCFA production, *Bifidobacterium* enhances gut barrier function, reducing endotoxin translocation from entering the bloodstream. The reduction in hepatic inflammation is further supported by *Bifidobacterium’s* ability to enhance regulatory T cell (Treg) activity and increase the production of IL-10 and TGF-*β*, thereby helping to suppress inflammatory responses (Round and Mazmanian, 2009; Kwon et al., 2010).

**Figure 1:**
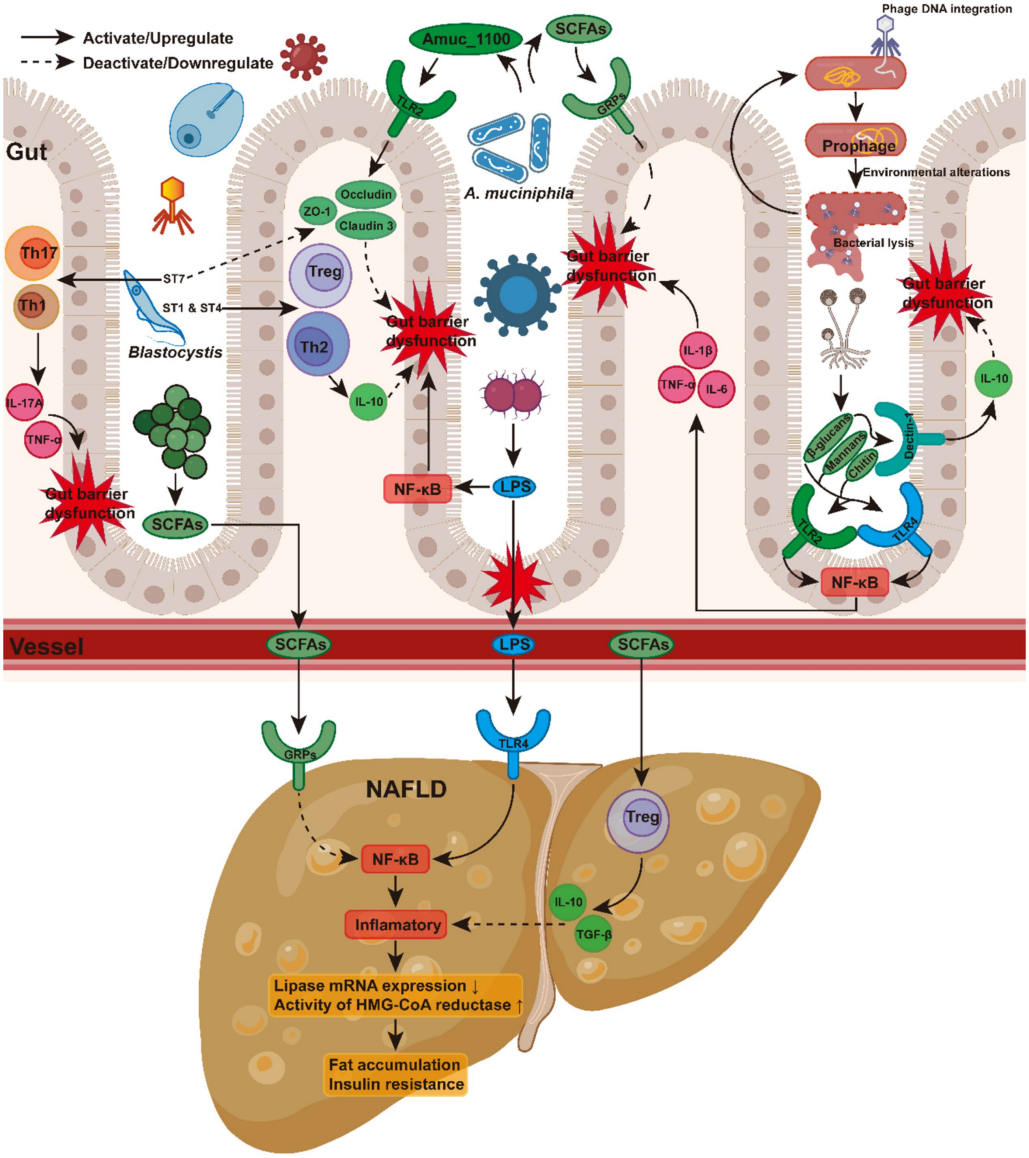
Mechanisms linking gut microbiota and liver function to NAFLD progression.

These microbial shifts indicate complex interactions in metabolic pathways, which exert their influence on NAFLD via several mechanisms. These include modulation of SCFA levels (Rau et al., 2018; Parada Venegas et al., 2019; Dai et al., 2020; Liu et al., 2022; Portincasa et al., 2022; Wang et al., 2022; Xiong et al., 2022), regulation of intestinal barrier integrity (Chang et al., 2014; Deleu et al., 2021; Forlano et al., 2022; Quesada-Vázquez et al., 2022; Liu et al., 2023; Fagundes et al., 2024), influence of host immune responses (Parada Venegas et al., 2019; Kim, 2021; Visekruna and Luu, 2021; Yao et al., 2022; Zhang Z. et al., 2022; Sarkar et al., 2023), and alteration of bile acid metabolism (Mouzaki et al., 2016; Appleby et al., 2019; Gottlieb and Canbay, 2019; Collins et al., 2023; Figure 1). However, as these complex biological pathways have been comprehensively reviewed elsewhere (Aron-Wisnewsky et al., 2020), we will not discuss them in detail here.

These findings highlight the critical role of gut bacteria in shaping NAFLD pathogenesis, influencing a range of biological processes. The bacterial composition within the gut has substantial effects on various aspects of NAFLD, including the regulation of metabolic pathways, immune modulation, and gut barrier integrity (Aron-Wisnewsky et al., 2020).

Nonetheless, our understanding of the role of bacteria in NAFLD remains incomplete. In addition to bacteria, other microbial components—including viruses, fungi, and parasites—contribute to this complex narrative (Iliev and Cadwell, 2021). The roles of these additional microbial elements in the development of NAFLD introduce further complexity. Investigating their specific contributions presents a promising opportunity to advance our understanding of NAFLD progression. This review seeks to elucidate these understudied aspects, potentially identifying novel therapeutic targets for the managing and preventing NAFLD through comprehensive modulation of the microbiota. Continued exploration of these multifactorial interactions holds the potential to uncover innovative interventions, benefiting those at risk of or currently suffering from NAFLD.

### Decoding the gut virome: bacteriophages, eukaryotic viruses, and the unexplored interactions in NAFLD pathogenesis

The gut virome—which comprises a diverse range of viruses, including eukaryotic viruses, bacteriophages and archaeal viruses acquired from diet and environmental factors—is a fundamental component of the gut microbiome. Its presence plays a pivotal role in regulating the balance of the human gut microbiota. The virome significantly influences the structure and functionality of the microbiome, affecting health and disease states. Research highlights the virome’s critical role in modulating the dynamics and functions of the gut microbiota, with implications for overall human health (Liang and Bushman, 2021). A summary of the key points is provided in Table 1.

**Table 1:** Comprehensive summary of gut virome and NAFLD interactions.

Bacteriophages	- Dominant in the gut microbiome (Sutton and Hill, 2019). - Lytic life cycle involves bursting bacterial cells (Young, 2014). - Molecular components of lytic bacteriophages (lysins, depolymerases, tail proteins) shape gut microbiota (Fischetti, 2008; Dunstan et al., 2021; Filik et al., 2022; Rothschild-Rodriguez et al., 2022; Alrafaie and Stafford, 2023; Wu et al., 2023). - Lysogenic bacteriophages integrate genetic material into the host, remaining dormant (Howard-Varona et al., 2017; Carey et al., 2019; Zuppi et al., 2021). - Fecal virome transplantation (FVT) in mice fed a high-fat diet shows benefits, weakened by antibiotics, highlighting the importance of phage-bacteria interactions in FVT effects (He et al., 2018; Rasmussen et al., 2020).
Human eukaryotic viruses	- Rotavirus infection reduces intestinal permeability during diarrhea, impacting gut health (Rasmussen et al., 2020). - Potential link between Norovirus infection and liver dysfunction, highlighting the need for comprehensive research on viral impact in NAFLD (Nakajima et al., 2012).
Mycovirus and parasitic virus	- Fungal and viral components influence the immune system, suggesting protective roles against inflammatory bowel disease (Iliev and Cadwell, 2021). - Gap in research on the specific interactions between eukaryotic viruses and gut fungi or parasites in NAFLD patients.

#### Bacteriophages

Bacteriophages—viruses that infect and replicate within bacteria—dominate the viral component of the gut microbiome (Sutton and Hill, 2019). However, due to the difficulty in identifying specific bacteriophage hosts, our understanding of their impact on NAFLD remains limited.

Bacteriophages follow two primary life cycles: lytic and lysogenic. In the lytic cycle, bacteriophages infect bacterial cells, causing the cells to burst and release newly formed phages (Young, 2014). Lytic bacteriophages use a “kill the winner” strategy, targeting dominant bacterial species while sparing less abundant ones. This strategy helps maintain microbial diversity and stability within the gut (Silveira and Rohwer, 2016; Sutcliffe et al., 2023). The effectiveness of this process is facilitated by molecular components within lytic bacteriophages, including lysins, depolymerases, and tail proteins (Filik et al., 2022; Rothschild-Rodriguez et al., 2022; Alrafaie and Stafford, 2023). Lysins degrade the bacterial cell wall, compromising its structural integrity (Fischetti, 2008). Depolymerases break down bacterial capsules, which shield certain bacteria, allowing phages to infect their hosts (Dunstan et al., 2021; Wu et al., 2023). Tail proteins enable phages to attach to the bacterial cell surface and inject viral genetic material (Taslem Mourosi et al., 2022). These mechanisms are central to understanding how lytic bacteriophages shape the gut microbiota and influence its composition, with broad implications for human health.

Lysogenic bacteriophages, by contrast, integrate their genetic material into the host genome using specialized enzymes called integrases (Howard-Varona et al., 2017; Carey et al., 2019). This process, known as lysogeny, results in the formation of a dormant prophage within the host cell, which can remain inactive for extended periods (Zuppi et al., 2021). Environmental factors such as unhealthy diet, chemical exposure, or stress can trigger prophage activation, initiating a switch to the lytic cycle (Pasechnek et al., 2020). This activation leads to bacterial cell lysis and the release of inflammatory compounds such as LPS, which potentially contributing to NAFLD development (Lin and Lin, 2019; Hsu et al., 2021).

In various ecosystems, bacteriophages display distinct reproductive strategies. Lytic phages, found in favorable environments, exhibit high virulence and cause rapid bacterial lysis. In contrast, temperate phages, prevalent in nutrient-poor environments, have lower virulence and smaller burst sizes, preferring to infect slow-growing bacterial populations. These phages often enter a lysogenic state, contributing to the co-evolution and stability of microbial communities. The ratio of lytic to temperate bacteriophages may serve as an indicator of gut microbiota health (Howard-Varona et al., 2017; Manrique et al., 2017).

NAFLD patients often exhibit unbalanced diets, characterized by high intake of saturated fats and low consumption of micronutrients (Yasutake et al., 2014; Lian et al., 2020). These dietary habits are also prevalent in urban populations, where they are associated with higher *Firmicutes*-to-*Bacteroidetes* ratios compared to rural counterparts (De Filippo et al., 2010). In both animal models and individuals with non-alcoholic steatohepatitis (NASH), high-fat diets have been linked to changes in the gut virome, including increased lytic phage activity, which further exacerbates gut dysbiosis (Zhu et al., 2013; He et al., 2018).

Fecal virome transplantation (FVT) is gaining recognition as a potential therapeutic strategy for correcting gut microbiota imbalances, particularly in metabolic disorders like NAFLD. Preclinical studies highlight FVT’s efficacy in improving metabolic parameters such as enhanced glucose tolerance and promoting weight reduction, especially in high-fat diet models. However, several challenges limit the clinical translation of FVT. Donor variability remains a major concern, as differences in virome composition can lead to inconsistent therapeutic outcomes. In addition, safety concerns regarding the possible transfer of pathogenic eukaryotic viruses necessitate rigorous donor screening, increasing both complexity and cost (Raeisi et al., 2023; Rasmussen et al., 2024; Zuppi et al., 2024). In one study, FVT in high-fat diet-fed mice significantly improved metabolic outcomes; however, the benefits were reduced when antibiotics were administered prior to the procedure, underscoring the critical role of phage-bacteria interactions in the therapeutic efficacy of FVT. Addressing these hurdles is vital for the reliable implementation of FVT as a standard treatment for NAFLD (Rasmussen et al., 2020).

Metagenomic analysis of the bacteriophage community in NAFLD patients has revealed notable findings (Lang et al., 2020). In patients with advanced liver fibrosis, phages infecting *Lactococcus* and *Leuconostoc* were markedly reduced, correlating with an increase in their bacterial hosts. Conversely, phages infecting *Streptococcus*, *Escherichia*, *Enterobacteria*, and *Lactobacillus* increased, while the abundance of these bacteria decreased. *Lactococcus* and *Leuconostoc*, commonly found in foods, can mitigate NAFLD through mechanisms such as modulating the gut microbiota, reducing LPS levels, producing SCFAs, regulating lipid metabolism, and alleviating inflammatory responses, ultimately aiding in weight reduction, decreasing liver fat content, and improving insulin resistance (Castro-Rodríguez et al., 2020; Naudin et al., 2020; Sun et al., 2020; Mendes et al., 2021; Lee et al., 2024). In contrast, *Streptococcus*, *Escherichia*, and *Enterobacteria* are associated with fatty liver disease (Munukka et al., 2014; Naka et al., 2014; Boursier et al., 2016; Aron-Wisnewsky et al., 2020). These shifts in bacteriophage abundance were inversely correlated with their bacterial hosts, highlighting the complexity of bacteriophage-bacteria interactions. This complexity necessitates further study, including the exploration of “viral dark matter”—unexplored viral components that may provide crucial insights into the role of bacteriophages in NAFLD pathogenesis.

#### Human eukaryotic viruses

Eukaryotic viruses, part of the gut virome, also significantly impact human health. Highly contagious viruses such as Norovirus and Rotavirus can cause gastrointestinal inflammation, leading to vomiting, diarrhea, nausea, abdominal pain, fever, and dehydration. These symptoms are particularly severe in young children and vulnerable adults. While bacterial diarrhea often increase intestinal permeability, Rotavirus has been shown to decrease permeability via 5-HT and neurotrophic factors (Hagbom et al., 2020).

Additionally, case reports suggest a potential link between Norovirus and liver dysfunction, though more research is needed to elucidate this relationship (Nakajima et al., 2012). Rotavirus infections are commonly associated with elevated liver transaminases, with about 20% of affected individuals showing increased ALT and AST levels (Nakajima et al., 2012). Both Norovirus (Pearson et al., 2019) and Rotavirus (Pane et al., 2014a,b; Pearson et al., 2019) have also been implicated in type 1 diabetes through their effects on the immune system. Type 1 diabetes primarily involves autoimmune destruction of insulin-secreting cells, leading to insulin deficiency. However, some individuals with type 1 diabetes may develop insulin resistance due to obesity or long-term insulin therapy, a condition sometimes referred to as “double diabetes,” which may increase the risk of developing NAFLD (Memaj and Jornayvaz, 2022).

This interconnection between enteric viral infections, liver dysfunction, and metabolic disorders underscores the need for comprehensive research into the interactions between gut viruses and systemic health. Understanding these mechanisms is crucial for developing preventive and therapeutic strategies.

#### Mycovirus and parasitic virus

The interaction between eukaryotic viruses, gut fungi, and parasites in the pathogenesis of NAFLD is an underexplored yet promising area of research. While fungal and viral components of the gut microbiome are known to influence immune modulation and metabolic processes, their specific roles in NAFLD remain largely undefined.

Fungal viruses can reduce the pathogenicity of their host fungi by inducing hypovirulence. These viruses spread horizontally through hyphal anastomosis and vertically via spore transmission, which can be either asexual or sexual. Some viruses can influence the metabolic pathways of fungal cells; for example, *Penicillium chrysogenum* virus (PcV) affects the expression of the host’s metabolic genes, leading to changes in glycolysis pathways and lipid metabolism. These metabolic alterations may impact the fungi’s responses to environmental stresses and the production of secondary metabolites, such as antibiotics (Ghabrial et al., 2015).

Specific fungi like *Candida* and *Saccharomyces* play a role in maintaining gut homeostasis and potentially protecting against inflammatory disorders (Iliev and Cadwell, 2021). However, these fungi may also contribute to dysbiosis in NAFLD patients, though the precise mechanisms remain unclear (Wu et al., 2021).

Eukaryotic viruses, which include those that infect fungi and parasites, could further complicate these interactions. Some studies suggest that parasitic protozoa, such as *Blastocystis* and *Giardia*, may alter the composition of the gut microbiota and exacerbate immune responses. This dysregulation could lead to compromised gut barrier function and increased susceptibility to metabolic diseases, including NAFLD (Wu et al., 2021).

Despite these insights, specific research on how eukaryotic viruses influence the gut’s fungal and parasitic communities in NAFLD patients remains minimal. The intricate relationships among viruses, fungi, and parasites in the gut microbiome are not well understood, particularly regarding their collective impact on NAFLD pathogenesis.

Therefore, expanding research in this area is crucial. Investigating these interactions could uncover critical insights into the mechanisms of NAFLD, offering new therapeutic targets through the modulation of these eukaryotic agents within the gut microbiome. Understanding this complex triad could help develop more comprehensive strategies for managing gut health and metabolic disorders like NAFLD.

### Fungal players in NAFLD: unraveling the complex interactions and potential impact of *Candida albicans*, *Saccharomyces cerevisiae*, and *Schizosaccharomyces pombe*

Fungi play a crucial role in the gut microbiota by interacting with bacteria through competition for niches, nutrient acquisition, and immune modulation (Peleg et al., 2010; Krüger et al., 2019; Pierce et al., 2021). These interactions significantly influence gut health, affecting immune responses and overall microbial balance. In NAFLD, fungal balance is often disrupted, with specific species influencing metabolic factors like insulin resistance and liver inflammation (Coyte and Rakoff-Nahoum, 2019). External factors, such as medications, diet, and environmental changes, can disturb this balance, leading to long-term shifts in the fungal community (Coyte and Rakoff-Nahoum, 2019). Fungal dysbiosis has been linked to NAFLD (Coyte and Rakoff-Nahoum, 2019; Demir et al., 2022), with some species associated with key metabolic dysfunctions (Szóstak et al., 2023).

Research on gut fungi in NAFLD is limited. Given the limited research on gut fungi in NAFLD, exploring related conditions like metabolic dysfunction-associated fatty liver disease (MAFLD) becomes important. The diagnostic criteria for NAFLD and MAFLD highlight distinct approaches to defining fatty liver disease, as requested by the reviewer. NAFLD is diagnosed by exclusion, with fatty liver defined as fat accumulation in more than 5% of hepatocytes in the absence of significant alcohol intake, viral hepatitis, or other specific liver diseases. In contrast, MAFLD is a newer concept that emphasizes metabolic dysfunction as the central factor in fatty liver disease. MAFLD’s diagnosis is based on the presence of metabolic dysfunction, regardless of other potential causes, and requires meeting at least one of the following criteria: overweight or obesity, type 2 diabetes, or metabolic dysregulation (e.g., abnormal blood pressure, lipid profile, or insulin resistance). Unlike NAFLD, MAFLD does not rely on ruling out other liver diseases or alcohol intake. Due to the substantial overlap in pathogenesis and clinical characteristics between NAFLD and MAFLD, researchers often integrate findings from both conditions to gain a comprehensive understanding of the pathophysiological processes involved in fatty liver disease (Eslam et al., 2020; Shiha et al., 2021). Specific fungal species associated with NAFLD, such as *Candida albicans* and *Mucor ambiguus* have been found to be enriched in patients with NAFLD, while *Saccharomyces cerevisiae* and *Schizosaccharomyces pombe* show lower abundance in the gut of NAFLD patients (Table 2).

**Table 2:** Alterations in gut fungal species in patients with NAFLD/MAFLD.

Condition	Fungal species (abundance change)	Potential role in liver metabolism	References
NAFLD	*Candida albicans* (↑)	Pro-inflammatory effects; activates Toll-like receptors (TLR2 and TLR4) and NF-κB pathway, leading to hepatic inflammation and triglyceride production.	You et al., (2021) and Demir et al. (2022)
	*Saccharomyces cerevisiae* (↑)	*Saccharomyces cerevisiae* contains *β*-glucans with immunomodulatory properties; may improve insulin resistance by activating anti-inflammatory pathways.	You et al. (2021) and Demir et al. (2022)
	*Saccharomyces cerevisiae* (↑)	*Saccharomyces cerevisiae* may have protective effects on liver metabolism.	You et al. (2021) and Demir et al. (2022)
MAFLD	*Mucor ambiguus* (↑)	Positively correlated with increased levels of total cholesterol, LDL-C, ALT, and AST; may trigger chronic low-grade inflammation via NF-κB pathway activation, affecting liver metabolism.	Niu et al. (2023)
	*Saccharomyces cerevisiae* (↓)	Reduction associated with higher GGT levels and fasting serum insulin; its cell wall components have anti-inflammatory effects; decrease may worsen insulin resistance.	Niu et al. (2023)
	*Schizosaccharomyces pombe* (↓)	Similar to *S. cerevisiae*; reduction may exacerbate insulin resistance and affect hepatic lipid metabolism.	Niu et al. (2023)
	*Saccharomyces paradoxus* (↓)	Potentially beneficial; reduction may negatively impact liver metabolism.	Niu et al. (2023)
	*Saccharomyces pastorianus* (↓)	Potential beneficial effects; reduction may be detrimental to liver metabolism.	Niu et al. (2023)
	*Purpureocillium lilacinum* (↓)	Less studied; reduction may impact gut health and liver metabolism; further research needed.	Niu et al. (2023)

Patients with MAFLD show a decrease in fungal alpha diversity compared to healthy individuals, indicating a reduction in the richness and abundance of fungal species in the gut. Specifically, MAFLD patients exhibit increased levels of *Mucor ambiguus* within their gut, which is positively linked with total cholesterol, low-density lipoprotein cholesterol (LDL-C), alanine aminotransferase (ALT), and aspartate aminotransferase (AST) (Niu et al., 2023). The proliferation of *Mucor ambiguus* may trigger a host immune response, leading to chronic low-grade inflammation. Chronic inflammation can influence cholesterol synthesis through activating the NF-κB pathway, which increases the production of pro-inflammatory cytokines including TNF-*α*, IL-1, and IL-6 (Brown and Gordon, 2001; Netea et al., 2006). Interestingly, a high-fiber diet resulted in a reduction in the presence of the pathogenic fungus *Mucor ambiguus* (Wang T. et al., 2023).

Conversely, *Saccharomyces cerevisiae* and *Schizosaccharomyces pombe* display lower quantities in the feces of MAFLD patients. The reduced presence of these fungi was associated with higher gamma-glutamyl transferase (GGT) levels and fasting serum insulin (Niu et al., 2023). The cell wall components of *Saccharomyces cerevisiae* primarily include *β*-glucans, mannans, and chitin, which possess immunomodulatory properties (Brown and Gordon, 2001). In particular, β-glucans can combine with Dectin-1 on intestinal immune cells, promoting the secretion of anti-inflammatory cytokines such as interleukin-10 (IL-10), thereby suppressing chronic inflammation (Brown and Gordon, 2001; Gantner et al., 2005; Dillon et al., 2006). These findings suggest a potential relationship between these fungi and the improvement of insulin resistance, as evaluated through the Homeostatic Model Assessment of Insulin Resistance (HOMA-IR), calculated using the formula:

HOMA−IR=Fasting Glucose×Fasting Insulin/22.5

Another study also revealed reduced gut fungal richness in NAFLD patients, although this reduction did not reach statistical significance. The log-ratio of *Candida albicans* to *Saccharomyces cerevisiae* significantly correlated with higher ALT and AST levels, more severe liver inflammation, and lower levels of high-density lipoprotein cholesterol (HDL-C) (Dillon et al., 2006). Furthermore, yeast species such as *Candida albicans*, *Pichia kudriavzevii*, and *Candida glabrata* in the gut contributed to triglyceride production in patients with NASH (Mbaye et al., 2022). This may be attributed to the cell wall components of *Candida albicans*, such as mannans and β-glucans, which activate host Toll-like receptors, particularly TLR2 and TLR4. This activation triggers the NF-κB pathway, ultimately resulting in the release of pro-inflammatory cytokines (Netea et al., 2002, 2006). In an animal study, the inclusion of *Candida albicans* in a high-fat diet led to an enrichment of *Akkermansia muciniphila*—a probiotic known to improve insulin resistance and typically reduced in patients with NAFLD (Tokuhara, 2021; Peroumal et al., 2022). An explanation for this phenomenon is that *Akkermansia muciniphila* is an obligate mucin-degrading bacterium that utilizes mucin in the intestinal mucus as a carbon and energy source (Belzer and de Vos, 2012). When the intestinal barrier is compromised, the metabolic environment of the mucus layer changes, providing more mucin resources for *Akkermansia muciniphila* growth. Consequently, when *Candida albicans* disrupts the intestinal barrier and thickens the mucus layer, the abundance of *Akkermansia muciniphila* may increase accordingly.

However, a study suggest a beneficial role of *Candida albicans*. It is involved in lipid metabolism due to the secretion of lipase, which can hydrolyze triglycerides (Macias-Paz et al., 2023).

An existing review delves into the intricate interactions among *Candida albicans*, the host, and the microbiota and how they impact the host’s health status (d’Enfert et al., 2021). These relationships are influenced by genetic and phenotypic variations among diverse *Candida albicans* isolates, the host’s immune capabilities, and the varying composition of the microbiota across different sections of the digestive tract.

The microbiota plays a pivotal role in supporting the host’s defense mechanisms against *Candida albicans* by producing butyrate. Butyrate stimulates the generation of antimicrobial peptide IL-37 and nitric oxide, both crucial in combating *Candida albicans*. Furthermore, specific members of the gut microbiota, such as *Blautia producta* and *Bacteroides thetaiotaomicron*, contribute significantly to host resilience against *Candida albicans* by promoting the production of IL-37. *Lactobacillus* stimulates the production of IL-22, instrumental in conferring resistance against the colonization of *Candida albicans*. IL-37, an anti-inflammatory cytokine, modulates immune responses by interacting with the IL-18 receptor and IL-1R8 (Su and Tao, 2021). This signaling cascade reduces excessive inflammation while enhancing the gut epithelial barrier function, which indirectly hinders *C. albicans* colonization by strengthening the physical and immunological defenses of the gut. IL-37 signaling also influences the activity of macrophages and dendritic cells, leading to increased production of antimicrobial peptides and enhancing the resilience of the host against fungal pathogens like *C. albicans* (Wang et al., 2024). In parallel, IL-22 plays a key role in gut epithelial defense by binding to the IL-22 receptor complex on intestinal epithelial cells (Zheng et al., 2008; Pickert et al., 2009). This stimulates the production of antimicrobial peptides, such as RegIIIβ and RegIIIγ, which directly target microbial pathogens (Zheng et al., 2008). IL-22 signaling also promotes epithelial cell proliferation and tight junction integrity, creating a robust barrier that resists fungal colonization (Pickert et al., 2009). These antimicrobial peptides not only exert direct fungistatic effects but also create an inhospitable environment for *C. albicans*, reducing its ability to establish a foothold in the gut. *Lacticaseibacillus rhamnosus* ATCC 53103 modulates the host’s pro-inflammatory response to *Candida albicans* by reducing the production of inflammatory substances such as IL-1α and granulocyte-macrophage colony-stimulating factor (GM-CSF), thus regulating the immune reaction within the host (d’Enfert et al., 2021).

Overall, these findings underscore the complex interactions between gut fungi and the development and progression of NAFLD. Further research is needed to comprehensively understand the relationship between fungi and NAFLD (Table 3).

**Table 3:** Summary of gut fungi and NAFLD interactions.

Overview of fungi-bacteria interactions	- Fungi are integral components of the gut microbiota, engaging in diverse interactions with bacteria, including occupying ecological niches, producing antibiotics, modulating host immune responses, and providing nutrients (Peleg et al., 2010; Krüger et al., 2019; Pierce et al., 2021). - The distinct ecological niches of fungi and bacteria influence each other’s survival strategies, thereby affecting the balance of the gut ecosystem (Coyte and Rakoff-Nahoum, 2019). - Antibiotics alter the balance between fungi and bacteria, with a more lasting impact on fungi, leading to long-term changes in the fungal community (Seelbinder et al., 2020).
Gut fungi and clinical parameters in NAFLD	- Reduced gut fungal richness in NAFLD patients is correlated with higher ALT and AST levels, more severe liver inflammation, and lower HDL-C levels (Demir et al., 2022). - Yeast species, including *Candida albicans*, promote triglyceride production in patients with NASH (Mbaye et al., 2022). - *Candida albicans* may lead to liver inflammation, but some studies suggest it has a beneficial role in lipid metabolism (Mbaye et al., 2022, 2022; Peroumal et al., 2022; Macias-Paz et al., 2023).
Complex interactions and potential therapeutic role	- The complex interactions among *Candida albicans*, the host, and the microbiota impact host health, involving genetic variations, immune capabilities, and the composition of the microbiota in different parts of the digestive tract (d’Enfert et al., 2021). - Microbiota such as *Blautia producta*, *Bacteroides thetaiotaomicron*, and *Lactobacillus* spp. play crucial roles in host defense against *Candida albicans* (d’Enfert et al., 2021). - The microbiota stimulate the production of antimicrobial peptides, regulating the host immune response against *Candida albicans* (d’Enfert et al., 2021).

### Parasites in the gut: exploring the potential impact of protozoans *Blastocystis*, *Giardia lamblia*, and *Entamoeba histolytica* on NAFLD and metabolic disorders

Parasites are organisms that live on or within a host, including various types such as protozoans and helminths. They engage in complex interactions with their hosts, potentially causing diseases but also modulating the host’s immune system functionality to a certain extent. The “hygiene hypothesis” posits that increased hygiene practices lead to reduced early-life exposure to parasites, including protozoans and helminths. This reduction may heighten susceptibility to allergies, asthma, and autoimmune diseases (Bach, 2018). Gut parasites are considered may play a role in human health because they activate the immune system to recognize and combat pathogens, thereby regulating both innate and adaptive immune pathways (Ianiro et al., 2022). A comprehensive review has illuminated the interaction between helminths and the gut microbiota, elucidating their impact on human health (Loke and Harris, 2023).

Protozoans are single-celled organisms that inhabit the human gut and can infect individuals at various life stages. Their prevalence varies globally, with higher infection rates observed in developing countries (Baron, 1996). For example, studies have reported that 46.8% of schoolchildren in the Merhabete District of Central Ethiopia and 42.3% in Sanandaj City, Iran, are infected with intestinal protozoans (Bahmani et al., 2017; Dagne and Alelign, 2021). In the Laghouat province of Southern Algeria, prevalence ranges from 13.3 to 17.3% (Sebaa et al., 2021). These high prevalence rates underscore the potential impact of protozoans on human health in these regions. The high prevalence of protozoan infections in regions like Laghouat Province may significantly impact human health by altering the gut microbiota, potentially increasing the risk of metabolic diseases. Protozoan infections can disrupt the balance of gut microbiota, leading to dysbiosis, which has been linked to metabolic disorders, including NAFLD. In regions with already limited healthcare resources, these disruptions may exacerbate health outcomes by affecting the gut-liver axis, contributing to liver inflammation, and impairing metabolic health. Thus, protozoan infections in these specific regions could have far-reaching implications not only for general health but also for metabolic diseases such as NAFLD.

Although protozoans significantly affect human health by modulating gut microbiota and host immunity, there is a scarcity of research exploring their role in diseases such as NAFLD and metabolic disorders. Given that protozoans can influence gut barrier function and metabolic processes (Ianiro et al., 2022), it is important to investigate their potential impact on metabolic health and liver diseases.

The most prevalent protozoans in the human gut are *Blastocystis*, *Giardia lamblia*, and *Entamoeba histolytica* (Chabé et al., 2017; Dagne and Alelign, 2021; von Huth et al., 2021). Therefore, we will explore the potential relationships between these protozoans and NAFLD in the following sections.

#### Blastocystis

Obesity and insulin resistance are closely linked to inflammation and oxidative stress originating from white adipose tissue (Caudet et al., 2022). These factors can lead to metabolic complications. Interestingly, the presence of *Blastocystis*, a common gut protozoan, has been inversely correlated with body mass index (BMI) (Andersen and Stensvold, 2016; Yañez et al., 2021).

The impact of *Blastocystis* on gut health appears to vary by subtype and region. For instance, studies in China found positive associations between various *Blastocystis* subtypes (STs) and beneficial bacteria like *Akkermansia* (Huang et al., 2024), while research in Belgium reported negative correlations between *Blastocystis* ST3 and ST4 and *Akkermansia* (Tito et al., 2019). In Singapore, infection with *Blastocystis* ST1 enriched beneficial bacteria such as *Alloprevotella* and *Akkermansia*, induced immune responses involving Th2 and Treg cells, and reduced the severity of DSS-induced colitis in mice (Deng et al., 2023b). Fecal microbiota transplantation from these mice conferred protection by inducing Treg cells and increasing SCFA production (Deng et al., 2023b). Similarly, ST4 colonization increased beneficial bacteria like *Clostridia vadinBB60* and *Lachnospiraceae NK4A136*. It enhanced SCFA production and upregulated the proportions of Foxp3+ and IL-10-producing CD4+ T cells. These changes contribute to anti-inflammatory responses and provide protection against DSS-induced colitis through Th2 responses and increased production of anti-inflammatory cytokine IL-10 (Deng et al., 2022). In contrast, infection with ST7 worsened colitis by increasing pathogenic bacteria and pro-inflammatory cytokines IL-17A and TNF-*α* in CD4+ T cells (Deng et al., 2023a). ST7 also reduced levels of *Bifidobacterium longum* and *lactobacilli*, which are important for maintaining gut barrier function (Yason et al., 2019). Additionally, it disrupts intestinal barrier integrity by degrading tight junction proteins such as occludin and ZO-1. This degradation allows more pathogens and toxins to cross the intestinal barrier, further intensifying the inflammatory response. Moreover, *Blastocystis* ST7 infection can activate Th17 and Th1 cells, leading to an increased release of pro-inflammatory cytokines, including IL-17A and TNF-*α* (Yason et al., 2019; Deng et al., 2023a).

*Blastocystis* subtypes exhibit distinct genomic characteristics, with significant differences in amino acid sequences, particularly in genes related to protein kinases and proteases, which may account for variations in their pathogenic potential (Gentekaki et al., 2017). Interestingly, the same *Blastocystis* subtype may interact differently with the gut microbiota in different regions, possibly due to adaptation through horizontal gene transfer from prokaryotic and eukaryotic sources. This gene acquisition allows *Blastocystis* to adapt to the gut environment by acquiring new functional genes involved in crucial metabolic pathways. These include carbohydrate utilization, anaerobic amino acid and nitrogen metabolism, oxidative stress resistance, and pH regulation (Eme et al., 2017). These acquired genes may influence the composition of the gut microbiota and its inflammatory status, highlighting the parasite’s adaptive nature and its complex interplay with the gut environment. Given these effects on gut microbiota composition, immune responses, and inflammation, *Blastocystis* may play a role in modulating metabolic disorders like NAFLD. However, further research is needed to elucidate these connections.

#### Giardia lamblia

*Giardia lamblia* infection affects host health by altering plasma serotonin levels, leading to intestinal hypersensitivity and increased susceptibility to bacterial translocation across the intestinal barrier (Halliez and Buret, 2015). It also induces changes in the expression of genes related to immune responses, cell cycle regulation, and apoptosis in intestinal epithelial cells, further contributing to cellular damage. Specifically, immune-related genes such as CXCL1, CXCL2, CXCL3, CCL2, and CCL20 are upregulated, promoting inflammation. Cell cycle disruption involves G1/S phase arrest due to DNA damage and oxidative stress. Apoptosis-related genes like Bax are upregulated, Bcl-2 is downregulated, and caspases (Caspase-9 and Caspase-3) are activated, leading to mitochondrial apoptosis (Cotton et al., 2015).

Following *Giardia lamblia* infection, there is a significant decrease in the activities of sucrase, maltase, and lactase—the enzymes responsible for breaking down carbohydrates to release monosaccharides (Solaymani-Mohammadi and Singer, 2011). This impairment in carbohydrate digestion leads to malabsorption and nutrient deficiencies. Additionally, infected children often exhibit widespread amino acid deficiencies and elevated levels of specific phenolic acids, which are byproducts of bacterial amino acid metabolism (Halliez and Buret, 2015). Furthermore, *G. lamblia* utilizes bile salts for growth, and its infection can increase bile secretion. This alteration changes the gut microbiota composition, increases unconjugated bile acids, enhances the expression of fibroblast growth factor 15, raises host energy expenditure, disrupts lipid metabolism, and ultimately reduces adipose tissue (Farthing et al., 1985). Collectively, these effects demonstrate that *G. lamblia* infection significantly impairs nutrient absorption and metabolism, contributing to the host’s nutritional deficiencies and metabolic disturbances.

Certain probiotics can mitigate the effects of *G. lamblia* infection. *Lactobacillus johnsonii* La1, which possesses bile salt hydrolase activity regulated by the bsh47 and bsh56 genes, can reduce *G. lamblia* activity (Riba et al., 2020). Moreover, pre-treatment with inactivated *Lacticaseibacillus rhamnosus* ATCC 53103 or its proteins has shown potential in reducing the severity and duration of *G. lamblia* infection by modulating the gut microbiota and mucosal immunity. Supplementation with live bacteria is even more effective, as observed in mouse studies (Allain et al., 2017; Shukla et al., 2020). These findings suggest that *G. lamblia* infection can impact host metabolism and gut barrier function, which may have implications for metabolic disorders such as NAFLD.

#### Entamoeba histolytica

Intestinal mucus secretion is essential for maintaining the mucosal barrier and protecting epithelial cells from pathogens. *Entamoeba histolytica* targets colonic MUC2 mucin, stimulating over-secretion from goblet cells (Leon-Coria et al., 2018). The vesicle-associated membrane protein 8 (VAMP8), present on mucin granules in goblet cells, regulates mucus secretion during defense against *E. histolytica*. Activation of VAMP8 is crucial for proper mucus production; its absence impairs mucus secretion. In VAMP8-deficient animals, *E. histolytica* infection compromises the mucosal barrier and increases the secretion of pro-inflammatory cytokines IL-1α, IL-1β, and TNF-α (Cornick et al., 2017).

*E. histolytica* trophozoites typically exist as commensal organisms in the colons of most infected individuals, with 90% remaining asymptomatic (Nagaraja and Ankri, 2019). In germ-free animals, amoeba infection does not cause disease, but pathogenicity is restored in the presence of gut microbiota, indicating that *E. histolytica*’s pathogenicity is closely related to the gut microbiota (Chadee et al., 1987). Symptomatic infections are often associated with gut microbiota dysbiosis, which reduces CXCR2 expression on neutrophils, hindering their migration to the intestine and worsening amoebic colitis (Watanabe et al., 2017; Leon-Coria et al., 2020).

Certain bacteria and their metabolites can reduce the pathogenicity of *E. histolytica*. Supplementation with *Clostridium scindens* enhances granulocyte-monocyte progenitor cells (GMPs) in the gut, increasing neutrophil populations and strengthening the immune response against *E. histolytica* (Watanabe et al., 2017). Transplantation of bone marrow from mice colonized with *C. scindens* into uninfected mice also increases intestinal neutrophils and provides protection against amoebic colitis (Burgess et al., 2020). Deoxycholate, a metabolite produced by *C. scindens* through bile acid metabolism, similarly increases GMPs and offers immune protection (Burgess et al., 2020). While *Enterobacteriaceae* induce *E. histolytica* genes associated with oxidative stress survival, this effect is not observed when amoebas are co-cultured with probiotics (Leon-Coria et al., 2020). *Lactobacillus acidophilus*, a probiotic in the human gut, reduces *E. histolytica* survival by 50% after a 2-h co-culture by promoting the oxidation of essential amoebic enzymes like pyruvate oxidoreductase, Gal/GalNAc lectin, and cysteine proteases (Sarid et al., 2022). The 2-h co-culture period demonstrates that significant enzyme oxidation and a reduction in parasite viability can be observed within this timeframe. However, while these results underscore the potential efficacy of *L. acidophilus* in rapidly impacting *E. histolytica* survival, further studies with extended co-culture durations and more complex, gut-like conditions are needed to confirm its long-term effects and clinical relevance. These interactions suggest that alterations in gut microbiota can influence the pathogenicity of *E. histolytica*, which may have implications for intestinal health and metabolic disorders such as NAFLD (Table 4).

**Table 4:** Interactions between protozoans and gut microbiota: key findings.

Blastocystis	- Inverse correlation with body mass index (Andersen and Stensvold, 2016; Yañez et al., 2021). - Regional variations in functionality. - Parasitism by subtypes (ST) 1 enriches beneficial bacteria, triggers immune responses, and reduces colitis severity in mice (Deng et al., 2023b). - ST4 colonization increases proportions of beneficial bacteria, short-chain fatty acid production, and protects against colitis (Deng et al., 2022). - ST7 infection exacerbates colitis severity by increasing pathogenic bacteria and pro-inflammatory cytokines (Deng et al., 2023a).
*Giardia lamblia*	- Alters plasma serotonin levels, leading to intestinal hypersensitivity (Halliez and Buret, 2015). - Increases vulnerability to bacterial penetration of the intestinal barrier (Halliez and Buret, 2015). - Infection results in increased bile secretion, enhanced expression of fibroblast growth factor 15, raised energy expenditure, and disruption of lipid metabolism (Farthing et al., 1985). - *Lactobacillus johnsonii* La1 with bile salt hydrolase-like activity diminishes *G. lamblia* activity (Riba et al., 2020). - Pre-treatment with *Lacticaseibacillus rhamnosus* ATCC 53103 or its proteins alleviates severity and duration of *G. lamblia* disease (Allain et al., 2017; Shukla et al., 2020).
*Entamoeba histolytica*	- Targets colonic MUC2 mucin, stimulating over-secretion of goblet cells (Leon-Coria et al., 2018). - Impacts vesicle SNARE VAMP8, crucial for proper mucus secretion (Cornick et al., 2017). - Infection compromises mucosal barrier and triggers increased secretion of pro-inflammatory cytokines (Cornick et al., 2017). - Symptomatic infections associated with gut microbiota dysbiosis (Leon-Coria et al., 2020). - *Lactobacillus acidophilus* reduces *E. histolytica* survival by triggering oxidation of crucial amebic enzymes (Sarid et al., 2022).

### Future perspectives and research gaps

Despite significant advancements in elucidating the role of the gut microbiome in the pathogenesis of NAFLD, several research gaps persist that warrant further investigation. Firstly, while numerous studies have highlighted the impact of bacterial dysbiosis on NAFLD, the contributions of other microbial communities—including viruses (especially bacteriophages), fungi, and parasites—remain underexplored. Comprehensive multi-omics approaches are necessary to understand the complex interactions among these microorganisms and their collective influence on hepatic metabolism and inflammation.

Secondly, the causal relationships between specific microbial alterations and NAFLD progression are not yet firmly established. Most existing studies are cross-sectional, making it challenging to discern whether microbiota changes are a cause or consequence of NAFLD. Longitudinal cohort studies and well-designed interventional trials are essential to determine causality and to identify potential microbial biomarkers for early diagnosis and prognosis.

Thirdly, the mechanisms by which the gut microbiota modulate host metabolic pathways and immune responses in NAFLD are not fully elucidated. While pathways involving LPS-mediated activation of TLR4 and subsequent NF-κB signaling are recognized, other pathways—such as those involving SCFAs, bile acids, and microbial metabolites affecting regulatory T cells—require deeper exploration. Unraveling these mechanisms could reveal novel therapeutic targets and inform the development of microbiome-based interventions.

Moreover, individual variability in microbiota composition, influenced by genetics, diet, environment, and lifestyle factors, poses a challenge for translating findings into clinical practice. Personalized medicine approaches that account for these variables are needed to design effective prevention and treatment strategies.

Lastly, while therapeutic interventions like fecal microbiota transplantation (FMT), probiotics, and prebiotics show promise, their long-term efficacy and safety in NAFLD patients are not well-established. Rigorous clinical trials with standardized protocols are required to assess their therapeutic potential and to optimize dosing regimens.

In conclusion, addressing these research gaps through interdisciplinary and translational research will enhance our understanding of the gut-liver axis in NAFLD. Such efforts are crucial for the development of innovative microbiome-targeted therapies that could improve patient outcomes and reduce the global burden of NAFLD.

### Summary

This review assimilates extensive research focusing on the intricate associations between gut microbiota and NAFLD. It highlights the potential therapeutic impact of certain microorganisms on NAFLD management. The dynamic interrelationships among bacteria, viruses, fungi, parasites, and the host, including the modulation of microbiota pathogenicity and virulence, are crucial factors to consider in devising comprehensive strategies to combat NAFLD. Understanding and harnessing the complex interactions within the gut microbiome will be instrumental in advancing our approach to address the challenges posed by NAFLD.
